# A Review of Rattlesnake Venoms

**DOI:** 10.3390/toxins16010002

**Published:** 2023-12-19

**Authors:** Phuc Phan, Anant Deshwal, Tyler Anthony McMahon, Matthew Slikas, Elodie Andrews, Brian Becker, Thallapuranam Krishnaswamy Suresh Kumar

**Affiliations:** 1 Department of Chemistry and Biochemistry, University of Arkansas, Fayetteville, AR 72701, USA; phucphan@uark.edu; 2 Department of Biology, Bradley University, Peoria, IL 61625, USA; tmcmahon@mail.bradley.edu (T.A.M.); mslikas@mail.bradley.edu (M.S.); eandrews@mail.bradley.edu (E.A.); 3 Department of Biological Sciences, University of Arkansas, Fayetteville, AR 72701, USA; bmbecker@uark.edu

**Keywords:** *Crotalus*, *Sistrurus*, toxin, venom composition, COVID-19, biomedical application

## Abstract

Venom components are invaluable in biomedical research owing to their specificity and potency. Many of these components exist in two genera of rattlesnakes, *Crotalus* and *Sistrurus*, with high toxicity and proteolytic activity variation. This review focuses on venom components within rattlesnakes, and offers a comparison and itemized list of factors dictating venom composition, as well as presenting their known characteristics, activities, and significant applications in biosciences. There are 64 families and subfamilies of proteins present in *Crotalus* and *Sistrurus* venom. Snake venom serine proteases (SVSP), snake venom metalloproteases (SVMP), and phospholipases A2 (PLA2) are the standard components in *Crotalus* and *Sistrurus* venom. Through this review, we highlight gaps in the knowledge of rattlesnake venom; there needs to be more information on the venom composition of three *Crotalus* species and one *Sistrurus* subspecies. We discuss the activity and importance of both major and minor components in biomedical research and drug development.

## 1. Introduction

Biomedical research on venom components is invaluable in developing therapeutic strategies owing to their specificity and potency [1]. Pharmacologically significant venomous snakes are mostly front-fanged and fall within three families, namely, *Atractaspidae*, *Elapidae*, and *Viperidae* [2], with a high amount of variability in the composition of their venoms. Such variation in biochemical composition can occur amongst closely related species and within species [3,4,5,6,7]. For example, geographical variation in the venom of pit vipers and adders has been correlated to their diet [4,5,8,9] or topographical features [10,11]. Venom composition variability can be intra-genus [12] or intraspecific [4]. Intraspecific venom compositions vary in their lethality (LD50), thus resulting in varying symptomatology and confused diagnosis or ineffective antivenom treatments, amongst other medical complications, during medical applications [4]. One example is during the treatment of *C. basiliscus* envenomation, which requires many different antivenoms to neutralize specific toxins in the varying venom compositions of just one species [13]. Deshwal et al. (2021) recently explored the variation in snake venom using meta-analysis to tease apart the relationships between different *Crotalus* and *Sisturus* venom components, which could be helpful in biotechnical and biomedical advancements [14]. The diversity observed in snake venom is often due to the recruitment strategy and duplication of toxin-encoding genes [15,16,17,18,19], followed by functional and structural diversification [1,20,21,22,23,24]. The phenomenon of venom diversification occurring at a high rate is supported by the hypothesis suggesting that venom is used for predation [4,22,25,26,27] and prey digestion [4]. Other studies have indicated that prey specificity is not the only driver of the venom diversification within *Crotalus* and *Sistrurus* [28], which is further supported by the studies demonstrating the differences in the venom composition between species despite having similar prey preferences [28,29,30].

Kocholaty et al. (1971) suggested that *Crotalidae*’s venom has the highest toxicity variation with a high proteolytic activity [31]. Rattlesnakes are within the subfamily *Crotalinae,* consisting of two genera, *Crotalus* and *Sistrurus*, with approximately thirty-six species [32]. Their habitats within the Americas range from southern Alberta, Saskatchewan, and southern British Columbia in Canada to central Argentina in a myriad of habitat types: from the Sonoran Desert of northwestern Mexico to alpine and cloud forests in central and southern Mexico [32]. This high variability in habitat type, altitude, associated diet types, and extensive geographical range allows rattlesnakes to have a high variability in their venom composition [14].

The high variability in range and distribution, venom composition, and activity provides ample opportunities to explore the venom components and their properties from biomedical and academic perspectives. However, numerous published works on rattlesnake venoms’ variability can sometimes hinder the understanding of critical relationships between different venom components [14]. Thus, an updated review of the rattlesnake venom system would be essential to highlight the gap in our knowledge and link the fundamental concepts of the rattlesnake venom system.

In this review, we discuss published research on rattlesnake venoms and their properties, as well as the biomedical applications of different toxin families from North, Central, and South America. Through this article, we aim to highlight data deficiencies in the current knowledge of venom compositions and applications of two families of rattlesnakes, *Crotalus* and *Sistrurus*, and delineate potential avenues for future research.

## 2. Rattlesnake Venom Comparison

**Figure 1:**
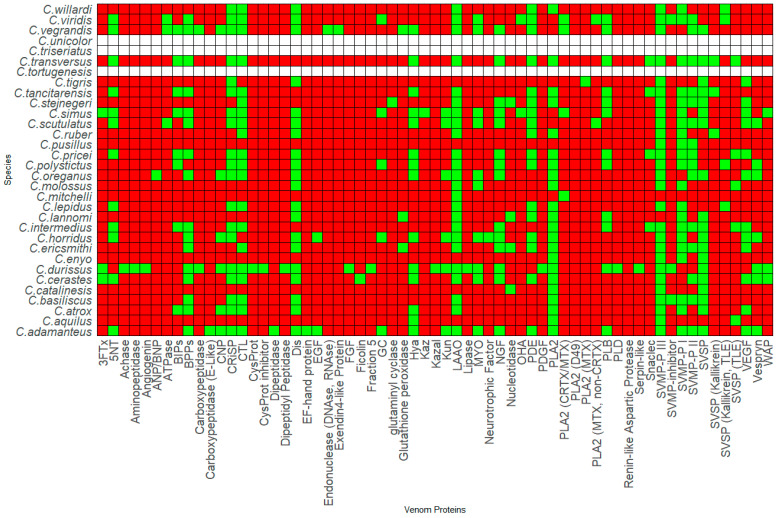
Presence-absence analysis of the genus *Crotalus* venom composition. The color of the box represents the presence (green), absence (red), and no information (white). The graph was generated using R. Abbreviations: Three-finger toxin (3FTx), 5′-nucleotidase (5′-NT), Acetylcholinesterase (Achase), Natriuretic peptide type A (ANP), Adenosine triphosphatase (ATPase), Bradykinin inhibitory peptide (BIP), Natriuretic peptide type B (BNP), Bradykinin potentiate peptide (BPP), C-type Lectins (CTL), Natriuretic peptide type C (CNP), Cysteine Protease (CYSPROT), Cysteine-rich secretory protein (CRiSP), Crotoxin (CRTX), Disintegrin (DIS), Epidermal growth factor (EGF), Fibroblast growth factor (FGF), Guanylyl cyclase (GC), Hyaluronidase (HYA), Kazal-type inhibitor (KAZAL), Kunitz-type inhibitor (KUN), L-amino acid oxidase (LAAO), Mojave toxin (MTX), Myotoxin (MYO), Nerve growth factor (NGF), Ohanin (OHA), Phosphodiesterase (PDE), Platelet-derived growth factor (PDGF), Phospholipase A2 (PLA2), Phospholipase B (PLB), Phospholipase D (PLD), Snake venom metalloprotease (SVMP), Snake venom serine protease (SVSP), Snake C-type Lectin (SNACLEC), Thrombin-like enzyme (TLE), Vascular endothelial growth factor (VEGF), Waparin (WAP).

**Figure 2:**
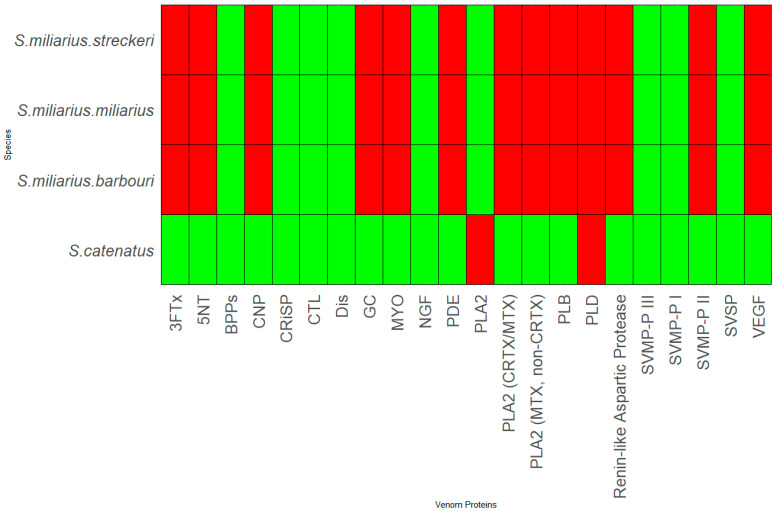
Presence-absence analysis of the genus *Sistrurus* venom composition. The color of the box represents the presence (green), absence (red), and no information (white). The graph was generated using R. Abbreviations: Three-finger toxin (3FTx), 5′-nucleotidase (5′-NT), Bradykinin potentiating peptide (BPP), C-type Lectins (CTL), Natriuretic peptide type C (CNP), Cysteine-rich secretory protein (CRiSP), Crotoxin (CRTX), Disintegrin (DIS), Guanylyl Cyclase (GC), Hyaluronidase (HYA), L-amino acid oxidase (LAAO), Myotoxin (MYO), Nerve growth factor (NGF), Phosphodiesterase (PDE), Phospholipase A2 (PLA2), Phospholipase B (PLB), Snake venom metalloproteinase (SVMP), Snake venom serine protease (SVSP), Vascular endothelial growth factor (VEGF).

Thirty-six species and sub-species within *Crotalus* and *Sistrurus*’ venom compositions were identified and documented through scientific articles that focused on transcriptomic and proteomic analyses of snake venoms. Sixty-four protein families and sub-families represent venom compositions for thirty-six rattlesnake species and subspecies. There are three *Crotalus* species for which we found no study documenting venom composition (Figure 1, Table S1). Sixty-three protein families and sub-families were present in *Crotalus* except for Renin-like Aspartic Protease, which is present in *Sistrurus catenatus* (Figure 1, Table S1). Venom composition was documented for all members in the *Sistrurus* family, and twenty-two protein families and sub-families, including Renin-like Aspartic Protease, were present in the venom of snakes within the genus *Sistrurus* (Figure 2, Table S2). Forty-two protein families and subfamilies in *Crotalus* venom were absent from all members of the *Sistrurus* family, which can be viewed in Figure 1 and Figure 2. Additionally, most venoms in both genera contain SVMP, SVSP, and PLA2.

*Crotalus* species offer a wide range of proteins due to their large genus, which results in a notable variation between species’ venom protein contents. The most common proteins in the venom compositions include CTL, Dis, CRiSP, SVSP, SVMP-P I-III, and LAAO. A high similarity is shown between *C. polystictus* and *C. Oreganus*, *C. Simus* and *C. Scutulatus*, and *C. viridis* and *C. vegrandis*. As demonstrated by Figure 1, the venom composition for *C. tortugenesis*, *C. unicolor*, and *C. triseriatus* needs to be quantified. 

Although fewer species exist in the genus *Sistrurus*, many proteins are found in these venom compositions. We see the same compounds in all three subspecies of the species *S. miliarius*. In contrast, the species *S. catenatus* contains a unique set of proteins along with most proteins found in the species *S. miliarius*.

Figure 1 and Figure 2 show the presence or absence of protein components found in rattlesnake venom. Additional information about the species’ venom, such as the percentage of proteins present, is not included in this report and must also be looked at.

The toxin families are enzymatic proteins such as L-amino acid oxidases (LAAO) [33,34,35,36], phosphodiesterase (PDE) [37,38,39], metalloproteinase (SVMP) [40,41,42], serine protease (SVSP) [34,43,44,45], and phospholipase (PLA2) [46,47,48,49]; or non-enzymatic proteins like myotoxin and its homologs [50,51,52,53], bradykinin-potentiating peptides and bradykinin-inhibitory peptides (BPPs & BIPs) [15,34,54,55], disintegrins (Dis) [33,34,44,56,57,58,59], cysteine-rich secretory proteins (CRiSPs) [33,44,52,60], and C-type lectins (CTL) [15,38,61,62]. Many minor venom components, such as LAAO and PDE, have not been explored for possible biomedical applications [17,63]. One possible reason for some protein families to receive lower attention in biomedical research is that the conventional classification system of venom components into major or minor components favors certain families over others [14].

## 3. Venom Protein Composition Patterns

Snake venom is affected by biotic and abiotic factors such as diet, age, habitat quality, range, and age [64]. Both genera, *Crotalus* and *Sistrurus*, are listed below, describing their characteristics.

### 3.1. Genus Crotalus

*C. adamanteus*

*Crotalus adamanteus* is found in southeastern coastal plains, expanding from southeastern North Carolina, westward along the coast to eastern Louisiana, southward to the Florida Keys, and is also found on many of the Atlantic and Gulf barrier islands [65]. Their habitat is primarily within dry sandy soil. *C. adamanteus* consumes mainly rabbits, squirrels, rats, and mice, and occasionally birds. The venom composition has only been tested in adults [65].

*C. aquilus*

*Crotalus aquilus* primarily inhabits the highlands north of the Transverse Volcanic Cordillera, specifically in the southern Mexican Plateau region. It has been documented in various Mexican states, including Veracruz, San Luis Potosí, Hidalgo, Querétaro, Guanajuato, Jalisco, and Michoacán. On the other hand, *C. triseriatus* has a more limited distribution, found in the high mountainous areas of the Transverse Volcanic Cordillera, located to the south of the range inhabited by *C. aquilus.* Species are abundant in cultivated agave fields but naturally found in the foothills and valleys of Mexico [66]. *C. aquilus* mostly consumes other snakes, rodents, and other large mammals [67], but more studies need to be carried out to see specific species. The juveniles of these species show similar components to their parts in potency, and hybrids of the species and *C. polystictus* have been shown to have high venom potency [68].

*C. atrox*

*Crotalus atrox* is found in western Arkansas, Oklahoma, Texas, New Mexico, Nevada, California, and Arizona. *Crotalus atrox* habitats include rocky substrate and bajadas, brush-covered plains, and desert foothills. The species primarily eat mammals, birds, lizards, and juvenile tortoises. The venom composition has been tested in adults, juveniles, and neonates; there was no comparison between age groups [69].

*C. basiliscus*

*Crotalus basiliscus* is found in western Mexico, in western Chihuahua, southern Sonora, Nayarit, Jalisco, Colima, and northwestern Michoacán. The species can be found in arid tropical thorn scrub and forest. They are also found in tropical semi-deciduous and deciduous forests. *Crotalus basiliscus* has been seen to feed on rodents [70]. The venom composition has only been tested in adults [71].

*C. catalinensis*

*Crotalus catalinensis* is found only on the Santa Catalina island in the Gulf of California. Santa Catalina Island is arid, with rocky soil and scattered cacti, brush, and trees. *Crotalus caralinensis* primarily preys on lizards, birds, and rodents [70]. No information was available on the age of tested snakes.

*C. cerastes*

*Crotalus cerastes* are found in southern California, Nevada, and northwestern Mexico in southwestern North America. The species are found in sandy dunes, washes, and flat desert lands. The primary diet consists of lizards, but they are also seen to eat mammals, birds, and snakes [72]. The venom composition has only been tested in adults [73].

*C. durissus*

*Crotalus durissus* is found in all territories of Brazil, with the highest occurrence in the north. The species can be found in open fields and dry, sandy, and stony areas [74]. *Crotalus durissus* consumes mostly rodents (75%), small marsupials (6%), reptiles (9%), and unidentified mammals (9%) [75]. The venom composition of adult and neonate snakes was tested, and newborns presented 60% less protein than the adult snakes [74].

*C. enyo*

*Crotalus enyo* is found in the southern two-thirds of the Baja California peninsula, from the southern tip to the San Quintin Valley. These snakes are found in arid lowlands and mountain slopes in rocky or vegetative areas where they can shelter under cover. *Crotalus enyo* has been seen to consume small rodents (83%) and lizards (17%), with little occurrence of consuming arthropods such as centipedes, specifically in the genus *Scolopendra* [76]. No information was provided on the age of the snakes tested.

*C. ericsmithi*

*C. ericsmithi* is found in Guerrero, Mexico [77]. These snakes inhabit pine forests [78]. There is no information on the diet of this species. The venom composition was only tested on adults [77].

*C. horridus*

*Crotalus horridus* is found in New England, Ontario, northern Florida, eastern Texas, and southeastern Minnesota. The species can be found in wet bottomlands. *C. horridus* mostly consumes rabbits, squirrels, rats, and mice, and occasionally birds, frogs, and snakes. The venom composition has only been tested in adults [65].

*C. lepidus*

*Crotalus lepidus* is found in Edwards Plateau, Mexico, along the Rio Grande River, southeastern New Mexico, and west Texas. It is commonly found in mountain ranges, plateaus, and rocky habitats [39]. The species consumes primarily lizards (55.4%), scolopendromorpha centipedes (28.3%), mammals (13.8%), birds (1.9), and snakes (0.6%) [79]. The venom composition has only been tested in adults [39].

*C. mitchelli*

*Crotalus mitchelli* is found in east-central California, southwestern Nevada, extreme southwestern Utah, and in the south through southern California and western Arizona to the southern tip of Baja California, Mexico [80]. The species is found in arid deserts and flatlands with brush, rocky outcrops, caves, and shrubland [81]. *Crotalus mitchelli* are seen to consume primarily rodents [82]. The venom composition has only been tested in adults [83,84,85].

*C. molossus*

*Crotalus molossus* is found in the southwestern areas of the United States through central and southern Mexican highlands [86]. The species can be found in rocky terrains such as talus slopes, canyons, and cliffs. *Crotalus molossus* consumes mainly rodents, lizards, and birds [86]. The venom composition has only been tested in adults [87].

*C. oreganus*

*Crotalus oreganus* can be found in central California and northern British Columbia [88]. The species are found in warm, arid regions and rocky outcrops [89]. *C. oreganus* consumes mammals (76.1%), lizards (14.8%), birds (4.5%), and amphibians (4.5%). The venom composition of both juveniles and adults has been tested, showing differences in compositions based on prey, with juvenile *C. oreganus* consuming more lizards and adults consuming more mammals [88].

*C. polystictus*

*Crotalus polystictus* can be found in central México, west of Mexico City [38]. The species can be found in native and modified grasslands. *C. polystictus* feeds primarily on rodents. The venom composition was tested and compared between adult, neonate, and juvenile snakes. Neonates were shown to have significantly lower levels of all proteins compared to the adult and juvenile snakes. In comparing adult and juvenile venom, there was no significant difference in the amount of SVMP, Kallikrein-like proteins, and PLA2 [38].

*C. pricei*

*Crotalus pricei* is found in southeastern Arizona, northeastern Sonora/western Chihuahua, Mexico, and Durango, Mexico [90]. They inhabit higher montane regions and the sky islands of Arizona. *C. pricei* primarily consumes lizards (68–87%), specifically the Yarrow’s spiny lizard and mammals (13–32%). The venom composition has been tested in adults only [90].

*C. ruber*

*Crotalus ruber* is found in Baja California, on the peninsula, and several associated islands [91]. The species can be found in rocky areas with vegetation and cacti. The diet of *C. ruber* consists mainly of mammals (91.6%), but also includes lizards (7.5%) and birds (0.9%). The venom composition has only been tested in adults [91].

*C. scutulatus*

*Crotalus scutulatus* inhabits the southwestern United States in the arid regions of the Mohave, Sonoran, and Chihuahuan Deserts. Its distribution across the southwestern United States includes southern California, southern Nevada, and extreme southwestern Utah down into western and southern Arizona, southwestern New Mexico, and trans-Pecos Texas [92]. The species can be found in high interior plains, temperate pine-oak, and mesquite-grassland vegetation areas. Lava beds have also been considered a suitable habitat. Their diet comprises numerous small mammals, lizards, and other small vertebrates. The venom composition has only been tested in adults [92].

*C. simus*

*Crotalus simus* is found in the Mexican states of Veracruz, Tabasco, Oaxaca, Chiapas, and Central America as far south as Costa Rica [93]. The species can be found in semi-arid tropical rainforests and coastal scrub forests. No information on diet was available. The adult and juvenile venom compositions have been tested, but comparison was not performed [93].

*C. tigris*

*Crotalus tigris* is found in Arizona, centered around Phoenix and Tucson. The species can be found in rocky desert uplands and shrubby lowlands. *C. tigris* primarily consumes mammals, reptiles, and birds [94]. There is no information on the age of the snakes whose venom composition was tested.

*C. vegrandis*

*Crotalus vegrandis* is primarily found in the central and western plateau regions in Venezuela’s Monagus and Anzoategui states. The species can be found in small, relictual forested areas. *C. vegrandis* consumes mostly small lizards [95]. There is no information on the age of the snakes whose venom composition was tested.

*C. viridis*

*Crotalus viridis* extends east of the Rocky Mountains to Iowa in the United States and south to northern Mexico, with the northern limit of their range extending into southern Saskatchewan and Alberta, Canada. More information is needed on the habitat of *C. viridis*. The species consumes primarily small mammals [96]. There is no information on the age of the snakes whose venom composition was tested.

*C. willardi*

*Crotalus willardi* is found in Zacatecas, Mexico, extending north into southeastern Arizona and southwestern New Mexico. The species can be found in mountains and mountainous areas [97]. *C. willardi* consumes lizards (51%), birds (28%), mammals (12%), and centipedes (8.4%) [98]. The venom composition has only been tested in adults [39].

*C. tortugenesis*

No information is available on their range, habitat, diet, or age differences in venom composition.

*C. stejnegeri*

*Crotalus stejnegeri* is found on the Pacific slope of Mexico [99]. The species can be found in rocky, flat-topped hills, tropical dry forests, and oak forests [99]. No information was available on the diet of *C. stejnegeri* or on the age of the snakes whose venom composition was tested.

*C. tancitarensis*

*Crotalus tancitarensis* is found in Cerro Tancítaro in Michoacán, México [100]. No information on the habitat was available. The species has been seen to consume primarily lizards. Adult and neonate venoms have been tested and showed a difference, with the adult venom containing two markers of PLA_2_ and the neonate venom containing neither [100].

*C. lannomi*

*Crotalus lannomi* is found in Puerto Los Mazos, Jalisco and Colima, Mexico [101]. The species can be found in tropical deciduous and oak forests. *C. lannomi* consumes lizards, arthropods, and plants. The venom composition has only been tested in adults [101].

*C. pusillus*

*Crotalus pusillus* is found in Sierra de Coalcoman of southwestern Michoacan, the Cordillera Volcanica of west central Michoacan, and adjacent southern Jalisco, Mexico [102]. The species can be found in limestone outcroppings in pine-oak forests [102]. No information on the diet of *C. pusillus*. There is no information on the age of the snakes whose venom composition was tested.

*C. transversus*

*Crotalus transversus* is found throughout Mexico [100,103]. The species can be found in the highlands. *C. transversus* consumes primarily lizards. The venom composition has only been tested in adults [100].

*C. triseriatus*

*Crotalus triseriatus* is found in central Mexico in the east-central Trans-Volcanic Mexican Belt [104]. The species can be found primarily in high-elevation mountains [104]. *C. triseriatus* consumes small rodents, pups of larger mammals, lizards, snakes, amphibians, insects, and centipedes [105]. There is no information on the age of the snakes whose venom composition was tested.

*C. unicolor*

*Crotalus unicolor* is found on the island of Aruba in the Dutch West Indies [106]. The species can be found on only 40% of the island in the steep hills, cliffs, and rocks [106]. No information on the diet of *C. unicolor* was available. There is no information on the age of the snakes whose venom composition was tested.

*C. intermedius*

*Crotalus intermedius* is found in Mexico [103]. The species can be found primarily in highlands at high elevations. *C. intermedius* consumes primarily lizards. The venom composition has been tested in adults and neonates, but a comparison was not made [103].

### 3.2. Genus Sistrurus

*S. catenatus*

*Sistrurus catenatus* is found in the Missouri Valley northeastward to Pennsylvania, New York, and southern Ontario. The species can be found in prairies, marshes, and swamps in spring and in higher and dryer locations in summer [107]. *S. catenatus* consumes frogs, birds, and young mammals [108]. There is no information on the age of the snakes whose venom composition was tested.

*S. miliarius miliarius*

There is no information on the range or habitat of *S. m. miliarius*. The species primarily consumes mammals and lizards [109]. There is no information on the age of the snakes whose venom composition was tested. The venom composition was only tested in adults [109].

*S. miliarius streckeri*

*Sistrurus miliarius streckeri* is found in Mississippi, extending east to South Carolina, south to the Florida Keys, and west to eastern Oklahoma and Texas [110]. The species can be found in flat hills, marshes, swamps, sandplains, and mixed forests [110]. Information was not available on the diet of the species. The venom composition has only been tested for adults [109].

*S. miliarius barbouri*

*Sistrurus miliarius barbouri* is found in southern Georgia, all of Florida, southern Alabama, and Mississippi [111]. The species is primarily found in open floodplains. *S. m. barbouri* primarily consumes lizards and frogs, sometimes eating small mammals. The venom compositions of adult and juvenile snakes were tested, but a comparison was not made [111].

### 3.3. Patterns in Venom Composition

**Figure 3:**
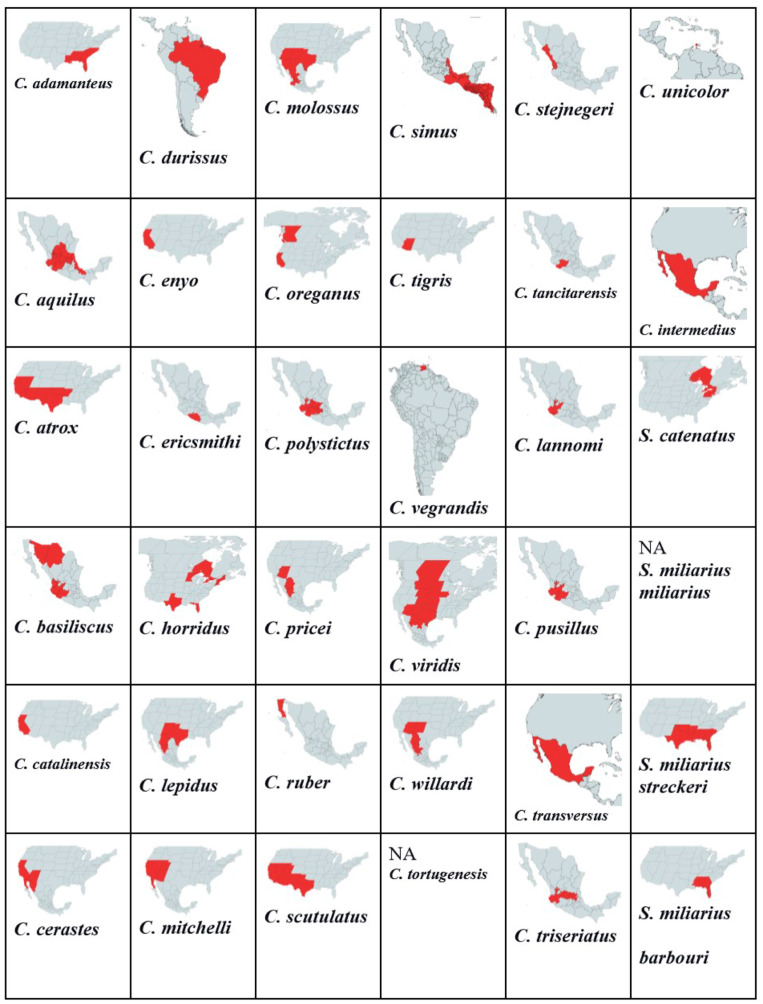
Range maps for each of the 36 *Crotalus* species. The red color in the map denotes the range of the species.

**Figure 4:**
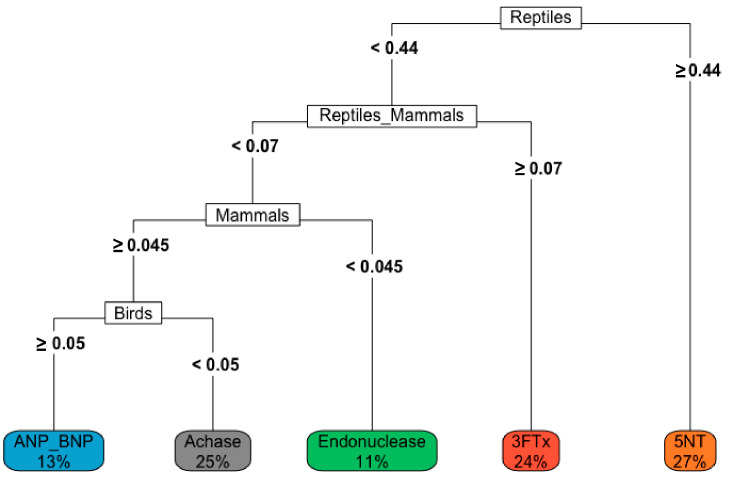
Decision tree demonstrating the role diet can play in the venom composition within rattlesnakes. We want to emphasize that the patterns demonstrated in this study in the venom composition are limited as most of the studies did not specify the rattlesnake’s source, age, and diet composition at the time of venom collection.

A total of 32 *Crotalus* species listed in Figure 1 range from Canada, the United States, Mexico, Brazil, Venezuela, and Costa Rica. We found that venom protein presence/absence patterns depended on range, distribution, diet, and habitat type. Within Mexico, *C. transversus*, *C. tancitarensis*, *C. pricei*, and *C. intermedius* found in Mexican highlands (Figure 3) differed in habitat preference from *C. ericsmithi*, *C. polystictus*, *C. simus*, *C. lannomi*, and *C. stejnegeri*, and *C. vegrandis* had a similar diet (Figure 4). These species had the following common proteins in their venom composition: 5′-NT, BIPs, BPPs, CRISP, CTL, LAAO, PLB, SNACLEC, SVMP P-III, and SVMP P-II. On the other hand, the difference in the diet composition of *C. aquilus,* also found in Mexico and in high elevations similar to that of *C. transversus, C. tancitarensis*, *C. pricei*, and *C. intermedius*, led to it having only two proteins (PLA2 and SVMP P-III) in common.

The decision tree based on the diet and venom protein composition shows how diet composition can affect the presence of a protein in snake venom, e.g., if a *Crotalus* spp. has the majority of its diet (≥44%), then there is 27% chance of 5′-NT being expressed in its venom (Figure 4).

**Figure 5:**
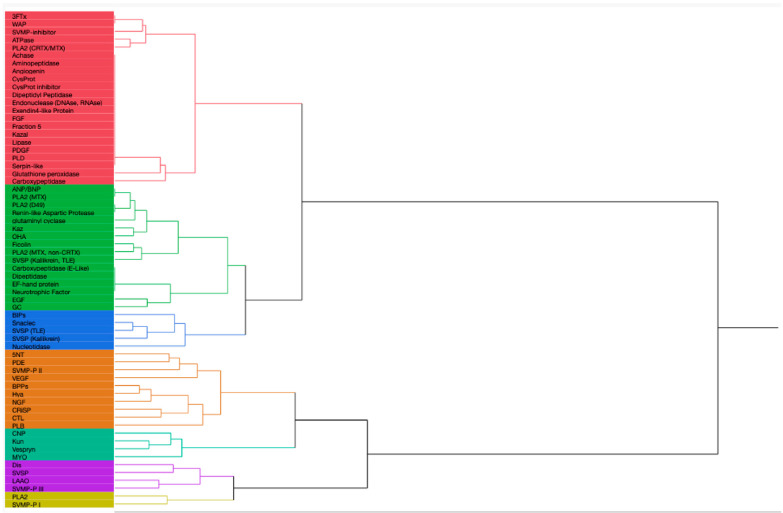
This dendrogram was created via the hierarchical clustering of proteins in rattlesnake venom by habitat type and distribution patterns. The proteins in red are most likely to be found in Central America. The proteins highlighted in green are most likely found in SW/S/W US and northern and southern Mexico. The proteins in blue are most likely to be found in SW/S/W US and south and western Mexico. The proteins in orange color are found throughout the range of rattlesnakes but are highly common in SW/S/W US and southern Mexico. Proteins highlighted by ocean green are mainly found in SE/SW/S/W US and northern Mexico. The proteins in purple color are present in high quantity throughout the rattlesnake range. The proteins in light green color are found in high abundance in central and southern Mexico. We want to emphasize that the patterns demonstrated in this study in the venom composition are limited as most of the studies did not specify the rattlesnake’s source, age, and diet composition at the time of venom collection.

Variation in habitat preference led to SNACLEC and SVMP-PI being present only in the venom of the following Mexican rattlesnakes: *C. transversus*, *C. tancitarensis*, *C. pricei*, and *C. intermedius*. Similarly, the venom composition of *Sistrurus catenatus* is quite different from the venom composition of *Sistrurus miliarius*. Figure 5 classifies proteins expressed in rattlesnake venom by habitat type and distribution patterns. No venom composition variation was seen between the three *Sistrurus miliarius* subspecies because most of the studies on their venom often purchased snake venom for analysis, leading to subtle variations by diet, range, habitat type, and age being lost [103]. We want to emphasize that the patterns demonstrated in this study in the venom composition are limited as most of the studies did not specify the rattlesnake’s source, age, and diet composition at the time of venom collection.

## 4. Venom Constituents and Biomedical Applications

Although venom components from 60 protein families and subgroups were identified, many are found through transcriptomics studies and have yet to be observed in proteomic works. In Section 4.1, we focus on some notable examples of venom components found in many rattlesnake species through proteomic analysis, and discuss their endophysiological targets and functions within snake venoms. Section 4.2 highlights potential avenues for future therapeutic developments of these venom constituents, focusing on the minor components of snake venom.

### 4.1. Venom Component Activities and Targets

#### 4.1.1. Disintegrins

It has been demonstrated that the DIS toxins derive from the protein family called A Disintegrin and Metaprotease (ADAM) in snake venoms [112,113]. Such ancestral relationships between cellular ADAMs containing the DIS-like domains and the DIS toxins have been studied in the literature [114,115]. DIS are small, non-enzymatic proteins [113,116,117] with members being classified into five groups of various sizes and numbers of disulfide bonds: (1) short, (2) medium, (3) long, (4) DIS-like domains in P-III SVMP, and (5) dimeric [57,112,114,118]. The short DIS has four disulfide bonds and around 49–51 residues [113,114]. At the same time, medium-sized DIS have approximately 70 residues and six disulfide bonds [118,119], such as mojastin 1 and 2 [57]. The third group is 100 amino acids long, with eight disulfide bridges [112,113]. As mentioned previously, many P-III SVMPs, like CamVMPII or jarhaggin, contain a DIS-like domain, around 100 amino acids in length, with eight disulfide bonds [116,120,121,122]. Apart from the first four groups, the fifth classification contains both homodimeric and heterodimeric DIS [44,123]. Many of them can bind to cell receptors on many cell types called integrins, which allow extracellular adhesions that implicate cell–cell and cell-matrix interactions [118] needed for cell proliferation, migration, and survival [117], and effectively inhibit the activity of integrins [112,113,119]. Many single-chain DIS have the active tripeptide residues of RGD, such as atroxatin and mojasin [56,57,112,113]. Exceptions in tripeptide residues can be seen with KGD, MVD, KTS, ECD, VGD, MGD, or WGD motifs in other DIS [112,117,118]. The conserved aspartate residue is proposed to be a specific binding site to the β subunits of integrins. At the same time, the other two amino acids handle the binding affinity to the α subunit [52]. This tripeptide is at the top of the loop protruding from the protein core [112], which the integrins would recognize as their ligands [112,118]. For example, the integrins αIIbβ3 recognized both RGD- and KGD-containing DIS [112], and a particular motif like RGD can bind to not just one integrin target [56,122]. However, such recognition differs amongst DIS containing the same tripeptide residues [112]. It has been observed that the dimeric type of DIS exhibits the highest level of diversity in their tripeptide motifs [112]. The inhibition of integrins from these toxins may help the distribution of other venom compounds throughout the tissues [52]. Other reported activities of DIS are anti-angiogenesis [124], the inhibition of platelet aggregation induced by several factors like thrombin, ADP, and collagen [57,117,118], and the inhibition of cancer cell migration and colonization [117,122,125,126,127,128,129,130]. These applications will be discussed in detail in Section 4.2.

#### 4.1.2. Cysteine-Rich Secretory Proteins

Cysteine-rich secretory proteins (CRiSPs) are a protein superfamily that has gained attention as a potential biopharmaceutical agent. CRiSPs are widely distributed in both genera of rattlesnakes [44,52,109,131,132,133] and have been crystalized and studied from snake venoms [134]. Members are single-chain peptides that contain approximately 230 amino acid residues [124,134], weigh 20–30 kDa [60,135], and contain a consistent pattern of 16 cysteines that are participating in internal disulfide linkages; hence the family name [60,134]. Modeled structures of CRiSPs revealed the two domains of the peptide: an N-terminal globular domain [124] and a C-terminal cysteine-rich domain, which contains 10 of the 16 cysteines [60,136], and a Zinc^2+^ binding motif [124,136]. However, reports of binding to Cd^2+^ have been noted [137]. The family possesses a wide array of biological interactions with ion channels with no precise main functions [136]: blocking ryanodine receptors [138], L-type calcium channels and/or potassium channels [60], and cyclic nucleotide-gated channels [136,139,140]. This binding can inhibit smooth muscle [141], which is its basal activity [115]. These interactions with different channels have been studied but require additional investigation [138,139]. Other intriguing activities from CRiSPs are anti-angiogenic activities [124], antiprotozoal activities (against *Trypanosomes* and *Leishmania*) [60], involvement within the inflammatory processes [138], and the inhibition of human umbilical vascular endothelial cell proliferation [131]. They appear to be non-toxic for mice and insects [60,124]. However, CRiSPs from *Philodryas patagoniensis* can produce mild myotoxicity when injected into gastrocnemius muscle without edema formation, inhibition of platelet aggregation, or hemorrhage [135]. The low toxicity to mammals and insects and the antiprotozoal activities could make CRiSPs a model for developing new pharmaceutical products [60].

#### 4.1.3. C-Type Lectins

Lectins are non-enzyme and non-immune proteins that can bind to carbohydrates. Snake venom is abundant in calcium-dependent (C-type) lectins, which can be grouped into two populations: the true C-type lectins (CTL) and the C-type lectin-like (snaclecs) [142,143]. Lectins are similar in glycan-binding specificities, determined by the proteins’ carbohydrate recognition domain (CRD) [144,145]. Such CTL domain has a Q-P-D tripeptides motif to determine the galactose specificities and is mediated by Ca^2+^ [146]. Snaclecs are often heterodimeric with CRD-like domains that cannot interact specifically with sugars [142,143]. In contrast, the true CTLs are homodimeric proteins with two identical disulfide-like subunits (around 15kD each), fully functional CRDs that bind carbohydrates and induce hemagglutination via surface glycoconjugates on erythrocytes [147,148]. Most CTLs’ members are galactoside-binding proteins that bind to the terminal galactoside residues using calcium [149]. Although only one rattlesnake lectin from *C. atrox* has been crystalized [150], many species within the *Crotalus* genus have been shown to obtain transcripts [65,123,151,152] and express CTLs [2,151,153]. Many lectins isolated from snake venoms share a high identity degree of 82–97% on the amino acid level, indicating a similar primary structure [149]. Secondary structures of CTLs seem to possess multiple β sheets and a couple of α helices [149,150]. Aside from the hemagglutination effects of CTL, some snake venom lectins can also induce mitogenesis of different cell types [154,155], while others cannot [156]. Similarly, some CTLs can induce platelet aggregation by a proposed mechanism of glycan recognitions on platelet surfaces that induces aggregation by CTLs [155,157], while others cannot [149,157]. Such variety in biological effects may be due to the carbohydrate specificity of lectins and surface receptors [154]. Other notable activities of CTLs are pro-inflammatory activity (Lomonte et al., 1990), renal toxicity [149], and some propitious activities such as antibacterial [158,159] and anti-tumoral activities in various cell lines [160,161,162], thus providing new avenues for researching this minor venom component.

#### 4.1.4. Bradykinin-Potentiate Peptides

Most toxins with low abundance in snake venom are vasoactive peptides [163], such as bradykinin-potentiate peptides (BPP). BPPs are described as pyroglutamyl proline-rich oligopeptides, 5–14 residues in length (~1 kDa), and with a conserved C-terminus rich in prolines [55,164,165,166]. As the name implies, BPPs can potentiate the bradykinin actions on various organs [167,168]. Such effects lead to hypotensive reactions in many organisms [165,168,169,170] due to the inhibition of bradykinin degradation [165,167,169]. The inhibition may also be accompanied by hyperpermeability of the blood vessels and loss of consciousness because of the potent hypotension [115]. Along with this inhibition, BPPs can also inhibit the conversion of angiotensin I to angiotensin II, the active form of angiotensin, creating a crucial pathway to develop angiotensin-converting enzyme (ACE) inhibitors due to the anti-hypertensive activities these effects induce [165,171,172]. The product called captopril, an ACE inhibitor for treating hypertension and heart failure, is a prime example of a commercialized drug based on snake venoms [164,171]. Recent reports have indicated that there is still much to learn from BPPs, such as the ability to distinguish between the N or C-terminal catalytic domains of ACE [173,174], or the discovery of ACE-independent mechanisms to reduce blood pressure [169,173]. Additionally, attempts to study the biogenesis of BPP have been conducted, and a report of a precursor polypeptide containing multiple sequences for BPP in tandem with a sequence of C-type natriuretic peptide (CNP) at the C-terminal was noted [55,164,175]. However, the processing mechanisms for this precursor are still elusive [55]. Thus, renewed interest in this vasoactive peptide has regained momentum with new BPPs isolated from many different snake species [165], many of which are from rattlesnakes [52,55,132,164,165,176].

#### 4.1.5. C-Type Natriuretic Peptides

Many precursors of BPPs contain another vasoactive peptide, the C-type natriuretic peptide family [55,164,175]. CNP is a member of the mammalian natriuretic peptide (NP) family that contains other subgroups (ANP and BNP), which have the C-terminus extension, and all are usually expressed in various tissues and organs of mammals [177,178,179,180]. CNPs are 22-amino-acid peptides with similar structures to ANP/BNP but differ from them genetically [181]. CNPs usually have an essential conserved ring core by forming an S-S linkage that contains 17 amino acid residues [179]. Snake venom CNPs can be around 30–39 amino acid residues in length, with a small molecular weight of around three kDa [182,183]. Members often bind to the guanylyl cyclase/natriuretic peptide transmembrane receptors (GC/NPR), which have three types (A, B, and C). NPR-C, which acts as a clearance receptor, has a high affinity to all NPs, while NPR-A has a high affinity to ANP/BNP, and NPR-B has a high affinity to CNP [177,178,179,180,184]. Upon binding to NPR-A/B, these peptides convert GTP into cGMP and release it as a second messenger for subsequent downstream pathways to enact its effects [181,184,185]. NPs influence motility in the gastrointestinal system. Specifically, NPs cause the relaxation of the esophagus, stomach, gallbladder, and colon [180,186]. Additionally, NPs produce potent hypotension in their prey during envenomation, contributing to a rapid loss of consciousness [186]. Although lacking the diuretic and natriuretic effects of ANP and BNP due to the absence of a C-terminus extension, CNP seems to have additional advantages [178] due to its less hypotensive effects [181,187], potent anti-proliferative activities, and collagen-suppressing properties [181,184]. Furthermore, CNP also benefits from its signaling receptors not being downregulated in the failing heart [178]. Thus, like BPPs, CNP has been extensively investigated as a therapeutic candidate for cardiovascular diseases [177,184,185,186]. DNP is the most studied NP, isolated from green mamba (*Dendroaspis angusticeps*) venom [177,179,181,188]. The result is a chimeric designer called CD-NP, a fusion of DNP from snake venom and human CNP [178,187], inheriting many novel and beneficial features from DNP and CNP [178,181,184,187]. With many notable activities, CD-NP, under the name Cenderitide, passed the phase I clinical trial [184]. Research avenues for CNPs are still open due to recent discoveries and successful isolations of new, unique CNPs from *Crotalus* [54,182,189] and other snake species [109,177,185,190].

#### 4.1.6. Nerve Growth Factors

Nerve growth factor (NGF) is among the least abundant toxins in snake venom [191,192,193,194,195,196]. Nevertheless, snake venom is considered a rich active source of this peptide [197,198]. Thus, snake venom provides much-needed accessibility to NGF compared to other growth factors and potentially lowers costs [197]. NGF is a peptide neutrophin (NT), important in maintaining nerve cells and repairing damaged cells [197,199,200]. Therefore, its existence within the venom arsenal is initially perplexing [200,201]. However, the family can also produce/enhance anaphylaxis [196,202] and induce mast cell degranulation [202], plasma extravasation, and histamine release [203], consequently leading to vascular permeability and tissue vulnerability, which aids toxin absorption and diffusion [201,203]. Additionally, this neuropeptide has a variety of non-toxic, ancillary biological activities: wound healing [200,204,205], effect on cartilage metabolism and chondrogenic differentiation [197], inhibition of metalloprotease-mediated degradation [200,201], involvement in inflammatory sites [200,205], and chemotherapy-induced neuropathy [206]. The protein can be isolated as a high molecular weight complex called 7S with 130 kDa in molecular weight composed of 3 subunits: α, β, and γ [195,207]. However, the β subunit (2.5S NGF) is the sole player in the neurotrophic activity of NGF [207]. It has two receptors. First is the tropomyosin kinase receptor A (TrkA), with high and specific affinity [194,208], triggers the MAPK, ERK, and PI3K/AKT cytosolic/endosomal pathways [197,208], leading to proliferation arrest and the induction of differentiation in neuronal cells [207]. Second is the p75 pan-NT receptor (p75NTR) [194,197], with similar affinity but not specificity, is linked to cell apoptosis and growth arrest via the MAPK c-Jun N-terminal kinase pathways [209]. Interestingly, both NGF receptors have been observed to be expressed in tumors in the nervous system and are especially prevalent in breast cancer [198]. NGF can promote or suppress tumor growth depending on tumor types [194,203], with prominent examples of NGFs from cobra venom inhibiting the growth of Ehrlich’s adenocarcinoma in vivo [194,198,210], but proliferative activity on breast cancer cell line MCF-7 [203,210,211]. Lately, NGF has been found to have a link to human diseases, including Alzheimer’s disease [200,212]. Subsequent NGF therapy for this neurodegenerative illness in phase I clinical trials has been reported [191,200], as well as other neurological disorders (Parkinson’s disease, peripheral neuropathy, etc.) [191,206]. Although most research on NGF reported here is based on Cobra species, there are studies on NGF from many snake species [213], including both rattlesnake genera *Crotalus* [195,213,214] and *Sistrurus* [109,111,132], that have indicated potential routes of isolating this active neuropeptide that may resolve the conflicting results of purifying NGF from snake venoms [195].

#### 4.1.7. Kunitz-Type Serine Protease Inhibitors

Kutniz-type inhibitors are a group of serine protease inhibitors that are often found in Elapidae and Viperidae snakes. It is believed that they play a role in interfering with the blood coagulation cascade, thus affecting the prey’s homeostasis [215]. As shown in Figure 1, seven of the *Crotalus* species have been reported to have this component in their venom composition. These inhibitors have around 60 residues and bear structural similarities with aprotinin [216]. They are reported to interact with serine protease via an exposed loop in a canonical confirmation, with the P1 residue acting as the primary site [217]. The P1 site also determines the specificity and reactivity of KUN towards its serine proteases [218]. Although KUN members are not highly conserved in their amino acid sequences [217], their overall structural scaffold is conserved, with subtle variations in the binding regions that aid in the functional diversity of KUN [218]. Currently, the members in the KUN family are divided into two major subgroups: non-neurotoxin (i.e., trypsin and chymotrypsin inhibitors) and neurotoxin (potassium and calcium blockers) [217]. Zupunski et al. (2003) report that Viperidae snakes, which include the *Crotalus* and *Sistrurus* genera, only contain the non-neurotoxic KUN members [217].

Due to its ability to bind to serine proteases, KUN offers promising pharmaceutical applications. Textilinin-1, isolated from *Pseudonaja textilis*, is shown to be very specific against plasmin and is one of the examples of promising biomedical applications. Specifically, it is shown to be a very effective and specific anti-bleeding agent with fewer side effects when compared to other agents like Trasylol [219]. Another KUN called tenerplasminin-1, isolated from *Micrurus tener tener*, is said to be a potent antifibrinolytic agent [220]. Such agents can be crucial in treating hyper-fibrinolysis events and excessive bleedings during medical intervention caused by heat strokes, hypotension, dengue infection, etc. [220]. KUN is often listed as a minor component of snake venoms but may prove to be a promising therapeutic agent in various biomedical settings.

#### 4.1.8. Waprin

St Pierre et al. (2008) posited that the KUN and WAP families may have been derived from a common ancestral gene with subsequent duplication and diversification events [221]. Transcripts containing both KUN-WAP have been identified in *Sistrurus catenatus*, further corroborating the relationship between these two components [222]. WAP is first isolated from *Naja nigricollis*, called nawaprin, and structurally resembles whey acidic protein [223]. WAP members are around 50 residues in length with four conserved disulfide bridges [221]. Like KUN, WAP is sorted as a minor component which three *Crotalus* genera and one *Sistrurus* genus are reported to possess (Figure 1 and Figure 2). Unlike KUN, which has been studied relatively well, WAP’s venom function is poorly characterized and understood [221]. One previous work reported some selective dose-dependent antimicrobial activities of omwaprin, a WAP isolated from *Oxyuranus microlepidotus*, through membrane disruption mechanisms [224]. On the other hand, previously reported nawaprin does not show any antibacterial activity nor does it act as protease inhibitor, a role that whey acidic proteins usually fill [223,225]. Limited information about WAP’s physiological functions and potential biomedical applications may warrant further research.

#### 4.1.9. Snake Venom Metalloproteases

The prominent presence of SVMP within rattlesnakes’ venoms has been discussed intensively through the Type I/Type II venom profiling dichotomy in *Crotalus* [37,226] and within two species of the genus *Sistrurus* [15]. Not only is SVMP abundant within rattlesnake venoms, with around 11% to over 65% of total venom protein [15,120], but it is also an important protein family present in the general Viperidae snake venoms [120], thus often being referred to informally as one of the major toxins within the world of venoms along with phospholipases and neurotoxins [17]. A high abundance of this protein family is thought to perform generic killing and digestive functions that are not prey-specific [120]. The presence of SVMP across different snake species significantly contributes to several pathological effects on blood coagulation [226] and fibrinogenolysis [16,226], leading to severe bleeding, local and systematic hemorrhage [227], and tissue damage after minutes of injection [226,228]. In numerous venomous snakebites, prothrombin activation [120], apoptotic responses [229], factor X-activating inflammation [120], and necrosis [227] may also occur. The precise mechanism of these effects is still elusive despite recent attempts to unravel the pathological effects of this protein family [228]. However, clues about such processes are given through various studies [230,231]. Briefly, hemorrhagic SVMPs seem to target the basement membrane and surrounding endothelial extracellular matrix, weaken the capillary walls, and reduce the width of endothelial cells, ultimately forming gaps amongst the weakened walls for erythrocytes to flow through [228,232]. Additionally, some endothelial cells are shown to be swollen and forming large blebs [120]. Consequently, several other basement membrane proteins, such as laminin, nidogen, and type IV collagen, seem to be reduced [120]. The SVMP family is classified within the M12 reprolysin family of metalloproteinase and further divides into three groups: P-I, P-II, and P-III [228,232]. P-I SVMPs comprise only one zinc-binding metalloproteinase domain with the lowest molecular weights (20–30 kDa) among SVMP subgroups [120]. A P-II SVMP contains an additional DIS-like domain, which is often released after proteolytic action, along with the zinc-binding metalloproteinase domain, making the protein bigger in the 30–60 kDa range and being thought to diverge from the P-III class [120,232]. Lastly, a P-III SVMP usually has a molecular weight of around 60–100 kDa, containing both the abovementioned domains and an extra cysteine-rich domain [120]. Some subclass members of P-III SVMP may also be linked to C-type lectin-like subunits and belong to the obsolete P-IV class of SVMP [120,228]. It has been reported that the P-III class tends to have higher hemorrhagic activities than P-I due to their size and resistance to α2-M (alpha-2-Marcoglobulin enhances prothrombin activation and thrombin activation) compared to P-I [120,227]. Additionally, the non-catalytic ancillary domains of P-III, namely, the DIS-like and cysteine-rich domains, may play important roles in P-II’s original hemorrhagic and additional non-hemorrhagic biological activities [227,232]. The crystal structures of nine P-I SVMPs have been elucidated along with their activities [233,234]. A prominent example is adamalysin II from *Crotalus adamanteus* [235], composed of a single chain of SVMP that needs Zn^2+^ and Ca^2+^ as cofactors for biological activities. However, not all SVMPs need Ca^2+^ to operate [120,235]. Similarly, around seven P-III SVMP crystal structures were found [233]. The crystal structure provided for VAP2B, a *Crotalus atrox* P-III SVMP, did reveal a dynamic, modular architecture of the three domains within P-II SVMP with important intrinsic flexibility for fine-tuning substrate recognition and post-translational regulation [236]. This finding seems to correlate with recent studies on the differences between hemorrhagic and non-hemorrhagic SVMP due to backbone flexibility in specific surface regions of the protein [237]. Despite lacking hemorrhagic activities, these SVMPs can still induce vascular permeability, inflammatory cell migration, and pain [232]. Drawing from such insights, many unexplored aspects of SVMPs would need to be further explored despite the large body of existing literature [120,232].

#### 4.1.10. Snake Venom Serine Proteases

Along with SVMP, SVSP is also considered one of the dominant toxin families within snake venoms [2,232] and has been observed to be present in almost all vipers [2]. SVSP is categorized in the S1 family of serine proteases [232], or the trypsin-like family [123], weighing 26–67 kDa [232]. Its members have evolved from kallikrein-like serine proteases [43,123] with significant gene duplications about the venom production, producing many isoforms [123,238,239]. SVSP was shown to be quite pharmacologically versatile, with a wide array of effects through subtle structural changes (Segura et al., 2017), with some expressing multiple activities [222]. In contrast to SVMP, which usually induces hemorrhages through capillary vessel rupture, SVSP alters the hemostatic systems of the victim [95], induces edema [43], hyperalgesia [232], blood coagulation perturbations [123], fibrinolysis [95,240], and platelet aggregation [43], and alters the kallikrein–kinin systems [95,152], by acting primarily on plasma proteins such as fibrinogen [43] to produce lethal consequences for the victims [232]. Characterized SVSPs are single-chain glycoproteins [43,222], although exceptions such as heterodimeric SVSPs have been found in *Agkistrodon. b. brevicaudus* [238]. Members usually have three substrate-binding sites and a catalytic triad with 12 conserved cysteine residues for six disulfide bridges [43,240]. Additional cysteine residues are usually found in SVSP, along with three N-glycosylation sites, which have been thought to contribute to enzyme stability and selectivity [240]. Due to its resemblance to trypsin, chymotrypsin, and thrombin, the aforementioned catalytic triad (composed of His57, Asp102, and Ser 195) can catalyze the peptide bond cleavage in which histidine is a proton donor/acceptor and serine acts as a nucleophile [43]. SVSP can be inhibited by various synthetic and natural products, such as phenylmethylsulfonyl fluoride (PMSF) [241]. This family of proteins is generally multifunctional, with many different substrates that warrant further investigation [43].

#### 4.1.11. Phospholipases A_2_

One of the most diverse classes of esterase is the phospholipase A_2_ (PLA_2_) enzymes, which prefer cleaving glycerophospholipids [46,47,242]. In a study completed in 2022 by Rodrigues et al., the PLA_2_ enzyme family was found to be the most abundant family within the entire *C. durissus* venom composition [243]. While PLA_2_ concentration was similar within all *Crotalus* subspecies, there were still differences between the subspecies, with *C. d. durissus* having the highest concentration compared to *C. d. cumanensis*, *C. d. ruruima*, and *C. d. terrificus* [243]. This class of proteins is divided into six families and further classified from I to XVI with capital letters [244]. Rattlesnakes’ secretory PLA2 (sPLA2) toxin can be listed in group IIA within the family of sPLA_2_, while those of cobras and kraits are listed in group IA [242,244]. These proteins are small and stable, with many disulfide bonds that tend to bind to Ca^2+^, and are highly similar in structures and sequences [46,47,242]. Additionally, within the known PLA_2_ secreted in snakes’ venoms, members are generally categorized into two groups: (1) catalytically active (D49 variant) and (2) catalytically inactive homologs (K49 variant) [47]. The D49 variants retain the conserved aspartic acid residue at position 49 at the catalytic center, essential for Ca^2+^ binding [47,242]. In contrast, the inactive variants, K49 PLA_2_, seem to replace the aspartic acid residue with lysine, thus losing the ability to cleave phospholipids but still having intriguingly crucial activities [47,245], and appear to be a suitable target for pharmacological discoveries [245]. Another form of PLA_2_ is a heterodimeric complex named crotoxin (CRTX) from a non-enzymatic polypeptide called crotapotin (CA) and a basic PLA_2_ (CB) [246,247,248,249]. Apart from the usual roles in lipid metabolism and membrane modeling, the PLA_2_ family of snake toxins displays a diverse array of biological and toxicological functions, including cytotoxicity [46,47], edema forming [46,250], anticoagulant [250,251], antibacterial [245,249], anti-tumoral [249], myotoxicity [46,47], and neurotoxicity activities [247,248,250,252,253]. The PLA_2_ family essentially plays both roles of phospholipases and neurotoxins within the venom of rattlesnakes. PLA_2_ achieves significant and specific neurotoxic effects on the presynaptic action (β-neurotoxic) [151], inhibiting the release of acetylcholine desensitizing the nicotinic receptors, leading to paralysis [248]. Many of these effects, such as antibacterial and antitumor effects for serum therapy and cancer treatment, are vital to study [47].

#### 4.1.12. Myotoxins

The family of myotoxins in rattlesnakes induces the same paralysis effect as PLA2 but with a different method [50,254]. This family is believed to derive from a common antimicrobial peptide ancestor called β-defensin in snakes, platypuses, and lizards [255,256]. Within these snakes, this toxin family seems to be exclusively expressed in rattlesnake species, around 11 species of *Crotalus* and *Sistrurus catenatus* [257], with no sign of its expression in other species of *Viperidae*. Myotoxins comprise peptides with around 42 amino acids and six cysteine residues for three disulfide bonds, making up a tight B-sheet core [50,258,259]. Thus, they are generally low in molecular weight and are often essential peptides rich in lysine and amphipathic [254,255,260]. Although they are less abundant than other previously mentioned major toxins, they are regarded as a major toxic component for many rattlesnake species [255,261], accounting for up to 20% of total toxins [33,262], with occasional listing as a minor family [263]. Toxic effects are present in the early and late stages of venom exposure. Myotoxins can disrupt cardiac proteins and cause cascades to destroy cardiac cells, leading to structural damage to the heart. Vascular leakage, swollen muscle fibers, edema, myocytolytic necrosis, and high trophin levels (an indication of heart stress) are all noted in lab rat studies found when exposed to venom from *C. durissus terrificus* [264].

Interestingly, this family has various biological activities like membrane penetration [254,265], nuclear localization [266], anti-tumoral activity, anti-fungal and antimicrobial activity [95,255,261], and irreversible membrane depolarization [254]. Thus, mycotoxin often induces the paralysis and extension of the hind paws by acting on Na^+^ and K^+^ channels [254,255,260] and inducing skeletal muscle necrosis [50,254,267]. In contrast to the neurotoxic PLA_2_, myotoxin’s mechanism is considered non-enzymatic and acts extremely rapidly to limit prey escape through hind paw paralysis and death through diaphragm paralysis [50]. Myotoxin was observed to localize in the sarcoplasmic reticulum and bind to its two components, one of which is Ca^2+^-ATPase, calcium pump, and the other may be a modulator of this calcium pump, which leads to the inhibition of calcium influx into SR and may have partly explained the paralysis effect [267]. Unsurprisingly, myotoxin’s structure shares essentially no similarity with PLA_2_ [50,262].

Additionally, the structure of myotoxin crotamine with an αββ fold and three disulfide bonds [268] is characteristic of membrane-active peptides and defines its ability to penetrate proliferating active human and murine stem cells [265,268]. Combining this activity with a stable structure scaffold, crotamine is portrayed as a versatile agent that can penetrate cells and cross the blood–brain barrier, with many other myotoxins’ features like anti-cancer activities [215,269]. Previous work has shown that crotamine is a potential tumor inhibitor to melanomas in mouse models [269], and a template for a synthetic analogue to deliver anticancer compounds in mammalian cells selectively [270].

Furthermore, previous studies have shown that members of this toxin family, specifically crotamine, are expressed differently amongst individuals and populations of *Crotalus durissus* [33,271,272]. Although the family has been discovered for over 50 years, many of its activities have been discovered in recent decades, with several more potential therapeutic applications [273].

### 4.2. Biomedical Applications

Snake bites have become a severe medical problem, especially in the tropics and subtropics [15,62]. Within Mexico, 40% of reported snake bites are from rattlesnakes [83]. Additionally, in the US, 7000–8000 bites from reptiles are reported to the American Association of Poison Control Centers each year [44]. To further complicate the issue, many components have not yet been characterized [15,62]. Within rattlesnakes, venom component characterization has been plagued by disproportionate attention received by several species and subspecies for various reasons. For example, *S. miliarius streckeri* has received the least attention compared to other subspecies (Figure 2, Table S2).

Similarly, within *Crotalus*, there are three species with no documentation of venom profile (Figure 1, Table S1). High plasticity and variability in rattlesnake venoms [32] is another cause of the incomplete characterization. Within the same species of *Crotalus*, the ontogenetic variation and potency in venom components is more pronounced in different age groups and between sexes than in other venomous snakes [109,190].

Creating an antivenom for each variation is not feasible [274]. Creating antivenoms primarily involves immunizing animals (such as horses, sheep, and cows) to produce antibodies that target and neutralize specific venom proteins [275]. These antivenoms are widely accepted and representative of the region’s snake population [275]. Within antivenoms produced from the genus *Crotalus,* the concentration of crotoxin present in the venom administered to the animal can change the efficacy of the antivenom received [275]. However, due to the high plasticity and variability of venom components, the potential impact of venom on biomedical and pharmaceutical discoveries is monumental [15,58,59,124,253,276], especially concerning minor components.

**Figure 6:**
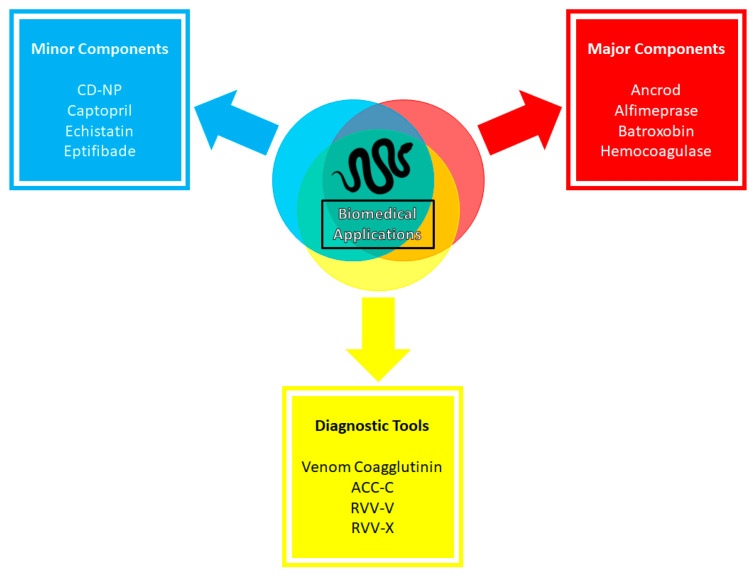
Biomedical applications of snake venoms. The graph demonstrates notable applications of snake venoms in biomedical research. Applications are separated into three groups: therapeutic drugs based on major components and minor components of snake venom, and diagnostic tools that are used in biomedical diagnosis and analysis.

In this review section, we aim to expand and/or discuss the applications of some of the venom components more in-depth, focusing on the minor components (Figure 6). NGF and natriuretic peptides are a few examples of the importance of minor components in biomedical research. NGF’s importance in biomedical research has been well documented over the years [277]. The administration of recombinant human NGF to treat various ophthalmologic diseases is well tolerated and has provided promising results [278]. Specifically, NGF has shown positive results for patients with dry eye diseases and improved symptoms in phase IIa clinical trials [279]. In glaucoma patients, NGF was safe and well tolerated, but produced no statistically significant results in the phase Ib clinical trial. However, the results showed strong trends towards significance in favor of NGF treatment groups and rendered the agent still relevant in treating glaucoma that warrants additional trials [280]. Recently, NGF has been reported to undergo a phase IV clinical trial in combination with electroacupuncture and rehabilitation training to aid ischemic stroke patients in improving brain functions and recovery period (NCT05231694). Moreover, ex vivo NGF-gene delivery therapy development for Alzheimer’s disease has been reported [191,200]. In summary, NGF was deemed safe, and no long-term adverse effects were observed in six patients with mild Alzheimer’s disease after 22 months of treatment. NGF also demonstrated improvement in the rate of cognitive decline and increases in cortical 18-fluorodeoxyglucose levels in PET scans after treatments, indicating broad increases of cortical glucose uptake and beneficially impacting cortical functions. The autopsy of one patient’s brain showed robust growth responses to NGF [281]. Subsequent reports showed that NGF treatment in Alzheimer’s patients is safe over extended periods. NGF expression lasts at least seven years; NGF-induced sprouting effects persist over ten years [282,283]. However, NGF-gene delivery therapy did not produce effective outcomes for Alzheimer’s disease patients in the phase II trial [284]. This is not to conclude that NGF therapy is ineffective, but only that the NGF-gene delivery had a limited spread and could not reach the cholinergic neurons of the basal forebrains [283,284]. Thus, NGF-gene delivery therapy is still seen as a promising treatment, with scheduled clinical trials in the future that utilize real-time magnetic resonance imaging for guidance and convection-enhanced delivery to improve targeting and spread [283]. Besides acting as a potent neurotrophic factor, NGF is another auspicious family for cancer treatment, but the results depend on the immune system [194,203]. Thus, it can be the next cancer cell treatment target [191,206].

Likewise, natriuretic peptides from snake venom have also seen notable developments. The making of chimeric peptides like CD-NP (Cenderitide), a fusion between snake venom DNP and human CNP, gives rise to new therapeutic agents with advantageous features of both original peptides: resistance to degradation, potent natriuretic and diuretic actions, minimal hypotensive effects, and inhibition of cardiac fibroblast proliferation [181,187]. Based on results from phase I of clinical trials, CD-NP may be critical in the treatment of cardiorenal diseases like acute cardiac failure and acute myocardial infarction [181]. This endeavor was terminated in 2017 [215], but it represents a clinically advanced first-in-class designer NP that advanced the field of therapeutic development for cardiovascular diseases [184].

Applications on minor venom components do not stop at proof-of-concept studies but progress and translate into commercial success. One of many examples of such success is applying bradykinin-potentiating peptides from *Bothrops jararaca* into captopril, an anti-hypertensive agent inhibiting angiotensin-converting enzymes [169,171,285]. From its first isolation as unstable BPP 5a in *Bothrops jararaca* venom in 1970 [167], BPPs have shaped the understanding of ACE inhibitors. Their structure and sequence were subsequently used to develop the first venom-based commercial antihypertensive drug of its class, captopril, that mimics the ACE binding motif of BPP5a [171]. Captopril was approved by the US Food and Drug Administration (FDA) in 1981 and by European Medicine Agencies (EMA) in 1984. Captopril became the milestone of snake venom-based therapeutic agents that saved countless lives [286]. More than just ACE inhibitors, BPPs have been reported to have neuroprotective activity in SH-SY5Y neuroblastoma cells against oxidative stress. BPPs assert their neuroprotective activities through the reduced reactive oxygen species generation and lipid peroxidation in H_2_O_2_-treated neuroblastoma cells [287]. Since post-heart-attack reperfusion is often accompanied by high oxidative stress and cardiac dysfunction, it is suggested that BPPs may be suitable for heart disease therapeutics due to their ability to reduce oxidative stress [288].

Furthermore, the family of DIS has also seen success as an approved therapeutic agents. Tirofiban is an antiplatelet drug approved by the FDA based on the toxin echistatin, a DIS member from *Echis carinatus* [286,289]. As this DIS can inhibit αIIBβ3 integrins by competing with fibrinogen, echistatin can inhibit the platelet aggregations’ final step [290,291]. Echistatin possesses an RGD motif and is selective to αIIBβ3 integrins within nanomolar affinity [292]. By enhancing the RGD motif with (S)-NHSO_2_*n*C_4_H_9_ extension, the new construct, called tirofiban, can interact with the exosite of the integrins and elevate the affinity and specificity of the design to αIIBβ3 [293]. Subsequently, the drug was approved for heart attack treatments by the FDA and EMA in 1998 and 1999, respectively [286,294]. Additionally, there was another FDA-approved DIS-based drug in 1998, based on barbourin toxin from *Sistrurus miliarius barbourin*, for acute coronary syndrome [295]. Barbourin is a KGD DIS, a variant from the commonly occurring RGD motif in other DIS members [296]. Using the structure of barbourin, a cyclized heptapeptide with a disulfide bridge was made, called eptifibatide (or integrilin) [295]. Such a cyclized structure helps eptifibatide to resist proteolysis while retaining the inhibitory actions towards αIIBβ3 integrin [297]. Furthermore, there is a recent surge of research in DIS’s inhibition of cancer cell migration and colonization, both in vivo and in vitro, and through recombinant vectors [125,127]. For example, recombinant rubistatin isolated from *Crotalus ruber ruber* has shown to be inhibitory towards SK-Mel-28 cancer cell lines [56]. Another DIS from *Crotalus durissus cumanesis*, cumanastatin-1, showed potent anti-platelet activity [126]. Likewise, colombistatin, a DIS isolated from *Bothrops colombiensis*, was reported to inhibit ADP-induced platelet aggregation and prevent cell adhesion and the migration of skin melanoma and human urinary cancer cells [119]. More recently, jararhagin-C, a DIS-like protein from *B. jararaca*, has shown wound healing and angiogenic activities in mouse models. Jararhagin-C also increases collagen deposition and induces the expression of pro-angiogenic cytokines, which renders the protein ideal for chronic wound treatments [298]. More examples of DIS members’ interactions with different human integrins and their functions in cancer are discussed in detail in Arruda Mecado et al. (2015) [125]. To sum up, these endeavors showed that minor components, especially molecular weight DIS from rattlesnakes, are promising candidates for many biomedical applications for various diseases [119,128,130].

Many other components of snake venom are still in preclinical phases and have yet to undergo clinical trials but demonstrate remarkable biological effects that could lead to practical biotechnological applications—for example, snake-venom lectins with their antibacterial and anti-tumoral effects via apoptosis [158,159,160,161]. Furthermore, CRiSPs, with their low toxicity to mammals and insects as well as their antiprotozoal activities and potent inhibitory effects on ion channels, could make CRiSPs a model for the development of new pharmaceutical products [60]. KUN may also possess some anti-tumoral activity. PIVL, isolated from *Macrovipera lebetina transmediterranea*, is shown to inhibit trypsin activity but also integrin αvβ3 activity without being cytotoxic; thus, it can inhibit human glioblastoma cell adhesion, migration, and invasion [299]. These protein families show the potential to be developed further as cancer treatments or antibiotic, antimicrobial, and anti-fungal agents [300].

Recent attempts to use and modify major groups of toxins have been made (Figure 6). One such example is the application of fibrolase, a recombinant thrombolytic SVMP from southern copperhead venom [301], to act as a clot lysis agent under the new name alfimeprase [302]. With the ability not to be inhibited by serine protease inhibitors [120,303], alfimeprase was observed to have a faster lysis activity than the plasminogen activator [304], but did not suffer from the potential of a systemic bleeding complication as the latter due to its intrinsic ability to be inhibited by α2-M [302]. Alfimeprase was in phase III clinical trials for treating patients with catheter occlusion and stroke but was discontinued in 2008 due to being ineffective and not meeting the trials’ endpoints of restoring the function of the occluded catheter within 15 min with a stringent *p*-value < 0.00125 [305]. Hemocoagulase, a combination of an SVMP and the SVSP batroxobin from *Bothrops atrox* or *Bothrops jararaca*, has also seen some commercial success. Hemocoagulase can activate factor X and convert fibrinogen to fibrin, thus increasing fibrin interaction with platelets to form clots and reduce bleeding time while improving the wound healing process [306]. It is commercially used as an injection and topical agent [307]. It is approved in Japan, India, and South Korea for internal and external hemorrhages [286].

Similarly, SVSP has been a prime candidate for treating certain hemostatic disorders. A good example is an SVSP batroxobin from *Bothrops atrox* [308]. This thrombin-like serine protease cleaves fibrinogen into fibrin and induces defibrinogenation, a quality exploited for thrombosis treatment [294,308,309]. However, it possesses some distinct features from thrombin: it only releases fibrinopeptide A after cleaving fibrinogen, unlike thrombin, which releases both fibrinopeptide A and B. Additionally, batroxobin binds to fibrin (nogen) with a higher affinity than thrombin and is more potent at triggering fibrin accretion [309]. Batroxobin, under the name Defibrilase, is available in China and Japan to treat several diseases and disfunctions, namely, deep vein thrombosis, ischemia caused by vascular occlusive diseases, pulmonary embolism, myocardial infarction, and acute cerebral infarction [43,286]. Like batroxbin, another SVSP was explored as a treatment for acute ischemic stroke due to its defibrinogenting activity, called ancrod or Viprinex [309,310]. This SVSP from *Calloselasma rhodostoma* can cleave fibrinogen to release fibrinopeptide A, reduce plasma viscosity, activate fibrinolysis, and improve microcirculatory flow [311,312]. However, ancrod did not show uniformly positive results, with one favorable profile for ischemic stroke patients treated within three hours of stroke onset and two no benefit profile outcomes for patients treated within six hours of stroke onset [311]. Vu et al. (2013) proposed that the procoagulant activity of thrombin-like SVSP contributes to microvascular thrombosis, thus complicating the treatment of these SVSP in ischemic stroke patients and producing no benefit results [309]. Others observed that ancrod induced fibrin formation, which resulted in cerebral microvascular occlusion and produced suboptimal effects on stroke patients [311]. Despite these results, ancrod has shown that SVSP and other major components are promising constructs that can be further optimized for the treatment of cardiovascular diseases.

Recently, reports of using PLA_2_ to test against SARS-Cov-2 showed promising effects and are gathering more interest [286]. Notably, heterodimer PLA_2_ HDP-2 from *Vipera nikolskii* was reported to have potent nanomolar virucidal activity [313]. Its phospholipolytic activity destroyed the viral envelope and inhibited glycoprotein-mediated virus–host fusion. HDP-2 can achieve this inhibition through binding with ACE2 and forming fairly stable complexes, thus blocking the interaction of glycoprotein S on SARS-CoV-2 with the host cell’s ACE2 receptor and preventing cell–cell fusion [313]. Besides virucidal activity, PLA_2_ also showed other notable effects. A 13-mer peptide pC-CoaTxII, a K49 PLA2 from *C. oreganus abyssus* venom, shows excellent antimicrobial activity against Gram-negative and -positive bacteria and multi-drug resistance clinical clones [245]. Crotoxin, a complex of PLA_2_ and crotapotin in *C. dyrissus terrificus*, shows antiviral effects in both its native form and the recombinant form [249]. Further investigations in the pharmaceutical utilization of PLA_2_ in virus treatments and cancer development are still ongoing with a promising outlook [84,253,285,314,315].

Even though crotamine has not undergone clinical trials, many of its beneficial activities have been reported with a promising therapeutic potential [286]. In modern antiviral applications, the protein crotamine in rattlesnake venom has been shown to decrease the replication of the SARS-CoV-2 virus. The D-enantiomer of the crotamine L-peptides (L-CDP) was shown to reduce viral replication and potentially target the viral protease essential for viral replication and protein cleavage [316]. Due to its cell-penetrating ability, especially for actively proliferating cells, crotamine is a potential antitumor agent [317,318]. Its inhibitory activity against melanoma has been reported without toxicity to healthy cells [269]. Due to its resistance to proteolysis, crotamine can be given orally [319]. The myotoxin peptides are even treated as templates for the development of cell labeling, chemical adjuvant [320], and drug delivery systems, such as the crotamine-derived NrTP class of cell-penetrating and nucleolar-targeting [265].

Snake venom components can also fulfill other roles as relevant diagnostic tools in biomedical research (Figure 6). For example, venom coagglutinin from *Bothrops jararaca* can enhance the affinity of the A1 domain of plasma von Willebrand factor for platelet receptor glycoprotein Ibα with broad-spectrum activity in various species [321]. With such characteristics, venom coagglutinin, named botrocetin, was developed as a diagnostic assay that can identify and quantify platelet-aggregating von Willebrand factor regardless of animal source [321,322]. Another prominent example is ACC-C, an SVSP from *Agkistrodon contortrix*, that activates plasma protein C [323]. Under the name Protac, it is used to determine the levels of protein C and protein S in blood [324]. Other components like SVSP RVV-V and SVMP RVV-X from *Daboia russelii* are also used to quantify factor V levels in plasma and factor X deficiency, respectively, and test the presence of lupus anticoagulant [325,326,327]. Such utilization of snake venoms is as relevant and crucial as venom-based therapeutic drugs, and should be the object of further investigation and development by academic experts and industry leaders.

Among the many successes of applying snake venoms as relevant biomedical drugs that have been discussed previously in this section, one can notice that most FDA-approved drugs belong to smaller non-enzymatic components. Indeed, prominent snake venom-based agents like captopril, tirofiban, and eptifibatide are all BPPs and DIS members. The large and/or enzymatic proteins from snake venoms that we have discussed so far, like alfimeprase and ancrod, are often unable to pass through clinical trials to be available in the market. There are exceptions like batroxobin and hemocoagulase, which are major snake venom components (an SVSP and an SVMP, respectively) and are commercially available. However, both are only approved for use in selected countries, leaving the US market with few therapeutic agents from major snake venom components. One explanation for such a phenomenon is the complexity and size of enzymatic components [328,329]. Although the industry now focuses on heterologous expression systems producing high molecular mass proteins, many obstacles in producing comparable three-dimensional folded products to native toxins remain [328]. For example, major enzymatic components require accurate cysteine bond formation and extensive post-translational modifications; both remain a challenge in heterologous expression production [328,329]. Moreover, prokaryotic hosts often cannot express active, mature forms of pharmaceutical proteins at high levels [328], which further limits the large-scale application process of these enzymatic components. Additional difficulties are also observed in assessing and alleviating the immunogenicity of the recombinant products in patients [329]. Lastly, many components in snake venoms assert their biological activities within the central nervous system. Thus, the ability to cross the blood–brain barrier becomes relevant during drug development. Despite the attractive functions that bigger and more structurally complex toxins provide, they are unable to cross the barrier efficiently. For example, NGF, a three-subunit 130 kDa neurotrophic protein that is essential for central cholinergic neuron survival, cannot cross the blood–brain barrier, while crotamine, a much smaller peptide with 42 residues, can cross the barrier and localize in brain cells [330,331]. Thus, NGF requires more strategies and modifications to cross the barrier and function effectively [331]. Combined, these factors affect the compatibility of large and/or enzymatic components of snake venom to be effective toxin-based biomedical candidates, as well as contributing to the reason why major snake venom components are not able to make it to the US pharmaceutical market. On the other hand, this phenomenon highlights the crucial roles of minor venom components as suitable candidates for pharmaceutical development [214]. Thus, the decision to choose and develop a particular component to be a therapeutical agent is advised to be specific, based on the structure and functions of the candidate, rather than automated [286]. Such differences in applicability of large and/or enzymatic proteins and smaller peptides also highlight the versatility and usefulness of minor components, and warrant more efforts from the pharmaceutical industry and academic professionals to study and develop these minor and often overlooked components [14].

## 5. Conclusions

Snake venoms, including rattlesnake venoms, are the most well characterized and applied in pharmaceutical development, owing to the large quantity of venom each individual produces compared to smaller venomous species [286]. Within their venoms, highly specific and potent toxin components, especially the minor components, are a goldmine for developing pharmaceutical applications. In this review, we compiled and consolidated the existing information on rattlesnake venom while highlighting the species/subspecies in which no venom profile exists. We also delineated the observed venom compositions of many species within rattlesnakes, in which SVSP, SVMP, and PLA_2_ are common in *Crotalus* venom, as described previously [2], as well as portraying the known characteristics of many toxin families expressed within rattlesnake venoms. A glimpse into the applied aspects of venom research shows promising avenues for future work not only on dominant families like proteases and neurotoxins but also on minor families like the growth factors. In this context, there is a need to characterize the toxin components of both *Crotalus* and *Sistrurus*, as well as minor components, to fill the gaps in knowledge in the field of toxicology and pharmaceutical interests [62].

## Data Availability

The data presented in this study are available in the manuscript and in supplementary materials.

## Supplementary Materials

The following supporting information can be downloaded at: https://www.mdpi.com/article/10.3390/toxins16010002/s1, Table S1. Detailed venom components of *Crotalus* genus (adapted from Deshwal et al., 2021 [14]). Table S2. Detailed venom components of *Sistrurus* genus (adapted from Deshwal et al., 2021 [14]). References [332,333,334,335,336,337,338,339,340,341,342,343,344,345,346,347,348,349,350,351,352,353,354,355,356,357,358,359,360,361,362,363,364,365,366,367,368,369,370,371,372,373,374,375,376,377,378,379,380,381,382,383,384,385,386,387,388,389,390,391,392,393,394,395,396,397,398,399,400,401,402,403,404,405,406] are cited in the supplementary materials.

## Abbreviations

3FTx—Three-finger toxin; 5′-NT—5′-nucleotidase; Achase—Acetylcholinesterase; ANP—Natriuretic peptide type A; ATPase—Adenosine triphosphatase; BIP—Bradykinin inhibitory peptide; BNP—Natriuretic peptide type B; BPP—Bradykinin potentiate peptide; CTL—C-type Lectins; CNP—Natriuretic peptide type C; CNP—Cysteine Protease; CRiSP—Cysteine-rich secretory protein; CRTX—Crotoxin; DIS—Disintegrin; EGF—Epidermal growth factor; FGF—Fibroblast growth factor; GC—Guanylyl cyclase; HYA—Hyaluronidase; KAZAL—Kazal-type inhibitor; KUN—Kunitz-type inhibitor; LAAO—L-amino acid oxidase; MTX—Mojave toxin; MYO—Myotoxin; NGF—Nerve growth factor; OHA—Ohanin; PDE—Phosphodiesterase; PDGF—Platelet-derived growth factor; PLA2—Phospholipase A2; PLB—Phospholipase B; PLD—Phospholipase D; SVMP—Snake venom metalloprotease; SVSP—Snake venom serine protease; TLE—Thrombin-like enzyme; VEGF—Vascular endothelial growth factor; WAP—Waparin.
