# Supplementary material for: A Review of Rattlesnake Venoms
Source: Toxins (Basel). 2023 Dec 19;16(1):2. doi: 10.3390/toxins16010002 (PMC10818703; doi:10.3390/toxins16010002)
Supplement: Supplementary file 1 [file toxins-16-00002-s001.zip › toxins-2722225-supplementary-done.pdf]

toxins

MDPI

Supplementary Materials: A Review of Rattlesnake Venoms

Phuc Phan, Anant Deshwal, Tyler Anthony McMahon, Matthew Slikas, Elodie Andrews, Brian Becker and Thallapuranam Krishnaswamy Suresh Kumar

Supplementary Materials: Table S1. Detailed venom components of *Crotalus* genus (adapted from Deshwal et al., 2021[14]). Table S2. Detailed venom components of *Sistrurus* genus (adapted from Deshwal et al., 2021[14]).

Table S1. Detailed venom components within *Crotalus* genus (adapted from Deshwal et al., 2021) [14].

Species	Venom components	Reference
C. adamanteus	5′NT, BPP, Carboxypeptidase (E-Like), CNP, CRiSP, CTL, Dipeptidase, DIS, EF-hand protein, EGF, GC, HYA, KUN, LAAO, MYO, NGF, PDE, PLA_2, PLB, SVMP-P I/II/III, SVSP, VEGF, Vespryn	[95,123,152,153,235,332–341]
C. aquilus	HYA, PLA_2, SVMP P-III, SVSP (TLE), LAAO, DIS	[49,62,68]
C. atrox	BIPs, BPPs, CNP, CRiSP, DIS, HYA, LAAO, CTL, PLA_2, SVMP P-I/II/III, SVSP, VEGF, Vespryn, 3FTx, MYO (crotamine), EGF, PLB, NGF	[34,117,152,229,230,236,263,340,342–346]
C. basiliscus	BPP, CRISP, CTL, DIS, LAAO, PLA_2 (CRTX, non-CRTX), SVMP P-I/II/III, SVMP-inhibitor, SVSP, MYO (crotamine)	[13,71,295,347–350]
C. catalinesis	SVSP, SVMP P-I/II/III, CTL, BIPs, LAAO, CRiSP, DIS, MYO, PDE, Nucleotidases, PLA_2	[83,351,352]
C. cerastes	3FTx, 5′NT, BPP, CRiSP, CTL, DIS, Ficolin, HYA, KUN, LAAO, MYO, NGF, PDE, PLA_2, SVMP P-II/III, SVSP, VEGF, Vespryn, WAP	[73,257,295,353,354]

C. durissus	3FTx, Achase, Aminopeptidase, Angiogenin, BPP, Carboxypeptidase, CNP, CRiSP, CTL, CYSPROT inhibitor,CYSPROT, Dipeptidyl Peptidase, DIS, FGF, Fraction 5, HYA, KAZA, KUN, LAAO, Lipase, MYO, NGF, PDGF, PLA_2 (non-CRTX, CRTX), PLB, PLD, Serpin-like, SVMP inhibitor, SVMP P-III, SVSP, VEGF, Vespryn, WAP	[33,54,165,247,248,260,271,285,349,355–364]
C. ericsmithi	SVSP, SVMP, DIS, CTL, LAAO, VEGF, Nucleotidase, NGF, PDE, BPP, GC, PLA_2	[77]
C. enyo	SVSP, SVMP P-I/III, PDE, PLA_2	[83,352]
C. horridus	5′-NT, BPP, CNP, CRiSP, DIS, EGF-like, GC, HYA, KUN, LAAO, MYO, Neurotrophic Factor, NGF, PDE, PLA_2 (canebrake), SVMP P-I/III, SVSP, VEGF, Vespryn	[65,116,153,257,339,348,365]
C. intermedius	SVMP P-II/III, SVSP (TLE, SNACLEC), PLA_2, Glutaminyl Cyclase, VEGF, DIS, BPPs, BIPs, CTL, CRiSP, LAAO, PLB, 5′-NT, NGF, NGF	[103]
C. lannomi	SVSP, SVMP, DIS, CTL, LAAO, VEGF, Nucleotidase, NGF, PDE, GC, PLA2	[77]
C. lepidus	5′NT, CRiSP, CTL, DIS, LAAO, PDE, PLA_2 (CRTX), SVMP-P-I/III, SVSP (TLE, Kallikrein)	[39,49,118,348,366,367]
C. mitchelli	LAAO, SVSP, PLA_2 (CRTX/MTX), PDE	[83-85,257,348,352]

C. molossus	DIS, LAAO, MYO (crotamine), PLA_2, SVMP P-I/III, SVSP (TLE),	[48,119,252,295,368–373]
C. oreganus	ANP/BNP, BPP, CNP, CRiSP, CTL, DIS, HYA, KUN, LAAO, MYO (crotamine), NGF, PLA2 (D49), PLA_2, SVMP P-II/III, SVSP, VEGF, Vespryn	[41,46,47,131,182,245,257,258,369,374–376]
C. polystictus	BIPs, CRiSPs, CTL, DIS, GC, HYA, LAAO, NGF, PDE, PLA_2, PLB, SVMP P-I/II/III, SVSP (Kallikrein, TLE), MYO, Vespryn	[38,62,68]
C. pusillus	SVMP	[377,378]
C. pricei	SVMP P-I/II/III, PLA_2, PDE, LAAO, SVSP (TLE), BIPs, BPPs, CTL, CRiSPs, HYA, PLB, 5′-NT, VEGF, NGF, DIS, SNACLEC	[90,103,377]
C. ruber	CTL, DIS, LAAO, PDE, PLA_2, SVMP P-I/III, SVSP (Kallikrein)	[37,83,352,379–384]
C. scutulatus	5′-NT, APase, BPPs, CRiSP, CTL, DIS, Hya, KUN, LAAO, MYO, NGF, PDE, PLA_2 (MTX, non-CRTX), SVMP P-I/II/III, SVSP, VEGF, Vespryn	[42,53,61,119,257,349,385–389]
C. simus	3FTX, 5′-NT, BIPs, BPPs, CRiSP, CTL, DIS, GC, HYA, KUN, KAZ, LAAO, MYO, NGF, OHA, PDE, PLA_2 (CRTX, non-CRTX), PLB, SVMP P-I/III, SVSP, VEGF, WAP	[45,58,129,151,349,390,391]

C. stejnegeri	SVSP, SVMP, CTL, LAAO, VEGF, Nucleotidase, NGF, PDE, GC, PLB, PLA2	[77]
C. tancitarensis	SVSP (TLE, Kallikrein), SVMP P-I/II/III, PDE, HYA, DIS, BIPs, BPPs, CTL, SNACLEC, 5′-NT, CriSP, LAAO, PLB, NGF, VEGF, PLA_2	[103]
C. tigris	CRiSP, DIS, PLA2 (MTX), SVMP P-III, SVSP, VEGF	[44,94,377,392,393]
C. transversus	SVSP (TLE, Kallikrein), SVMP P-I/II/III, PDE, HYA, DIS, CTL, BPP, BIP, CRiSP, Snaclec, LAAO, PLB, PLA_2, 5′-NT, VEGF, NGF	[103]
C. vegrandis	5′-NT, ATPase, BIP, BPP, Carboxypeptidase, CNP, CRiSP, CTL, DIS, Endonuclease (DNAse, RNAse), Exendin4-like Protein, Glutathione peroxidase, HYA, LAAO, MYO, NGF, PDE, PLA_2 (CRTX), PLB, SVMP P-II/III, SVSP	[95,253,394–398]
C. viridis	5′-NT, APase, BPP, CRiSP, CTL, DIS, GC, LAAO, MYO (crotamine), OHA, PDE, PLA_2 (CRTX, non-CRTX), PLB, SVMP inhibitor, SVMP P-I/II/III, SVSP (TLE, Kallikrein)	[50-52,257,399–403]
C. willardi	CRiSP, CTL, DIS, LAAO, PDE, PLA_2, SVMP P-I/III, SVSP (TLE, Kallikrein)	[39,377]
C. tortugenesis	N/A	
C. triseriatus	N/A	
C. unicolor	N/A	

Abbreviations: 3FTx - Three-finger toxin; 5′-NT - 5′-nucleotidase; Achase - Acetylcholinesterase; ANP -Natriuretic peptide type A; ATPase - Adenosine triphosphatase; BIP - Bradykinin inhibitory peptide; BNP - Natriuretic peptide type B; BPP - Bradykinin potentiate peptide; CTL - C-type Lectins; CNP - Natriuretic peptide type C; CNP - Cysteine Protease; CRiSP - Cysteine-rich secretory protein; CRTX - Crotoxin; DIS - Disintegrin; EGF - Epidermal growth factor; FGF -Fibroblast growth factor; GC - Guanylyl cyclase; HYA - Hyaluronidase; KAZAL - Kazal-type inhibitor; KUN - Kunitz-type inhibitor; LAAO - L-amino acid oxidase; MTX - Mojave toxin; MYO - Myotoxin,; NGF - Nerve growth factor; OHA - Ohanin; PDE - Phosphodiesterase; PDGF - Platelet-derived growth factor; PLA2 - Phospholipase A2; PLB - Phospholipase B; PLD - Phospholipase D; SVMP - Snake venom metalloprotease; SVSP - Snake venom serine protease; TLE - Thrombin-like enzyme; VEGF - Vascular endothelial growth factor; WAP - Waparin.

Table S2. Detailed venom components of Sistrurus genus (adapted from Deshwal et al., 2021) [14]

Species	Venom components	Reference
S. catenatus	3FTx, 5′-NT, BPP, CNP, CRiSP, CTL, DIS, GC, LAAO, MYO, NGF, PDE, PLA_2 (CRTX, non-CRTX), PLB, Renin-like Aspartic Protease, SVMP P-I/II/III, SVSP, VEGF	[109,190,222,238,274,365,404,405]
S. miliarius miliarius	BPP, CRiSP, CTL, DIS, NGF, PLA_2, SVMP P-I/III, SVMP-inhibitor, SVSP	[110]
S. miliarius streckeri	BPP, CRiSP, CTL, DIS, NGF, PLA_2, SVMP P-I/III, SVMP-inhibitor, SVSP	[110]
S. miliarius barbouri	BPP, CNP, CRiSP, DIS, PLA_2, SVMP P-I/III, SVSP	[15,109,110,132,190,274,365,406]

Abbreviations: 3FTx - Three-finger toxin; 5′-NT - 5′-nucleotidase; Achase - Acetylcholinesterase; ANP -Natriuretic peptide type A; ATPase - Adenosine triphosphatase; BIP - Bradykinin inhibitory peptide; BNP - Natriuretic peptide type B; BPP - Bradykinin potentiate peptide; CTL - C-type Lectins; CNP - Natriuretic peptide type C; CNP - Cysteine Protease; CRiSP - Cysteine-rich secretory protein; CRTX - Crotoxin; DIS - Disintegrin; EGF - Epidermal growth factor; FGF -Fibroblast growth factor; GC - Guanylyl cyclase; HYA - Hyaluronidase; KAZAL - Kazal-type inhibitor; KUN - Kunitz-type inhibitor; LAAO - L-amino acid oxidase; MTX - Mojave toxin; MYO - Myotoxin,; NGF - Nerve growth factor; OHA - Ohanin; PDE - Phosphodiesterase; PDGF - Platelet-derived growth factor; PLA2 - Phospholipase A2; PLB - Phospholipase B; PLD - Phospholipase D; SVMP - Snake venom metalloprotease; SVSP - Snake venom serine protease; TLE - Thrombin-like enzyme; VEGF - Vascular endothelial growth factor; WAP - Waparin.
